# Stanford Type A Aortic Dissection Manifesting as Acute Lower Limb Ischemia

**DOI:** 10.7759/cureus.98241

**Published:** 2025-12-01

**Authors:** Heloise Paccaud, Guérisse Fabien, Michael Beauprez, Quentin Vangyte

**Affiliations:** 1 Emergency Medicine, Erasmus Hospital, Brussels, BEL; 2 Emergency Medicine, CHU de Charleroi, Lodelinsart, BEL

**Keywords:** acute, acute lower limb ischaemia, aortic dissection, lower limb pain, stanford-a

## Abstract

Aortic dissection is a life-threatening emergency that requires prompt diagnosis. Clinical presentations vary and can be typical, atypical, or even asymptomatic. Aortic dissection manifesting solely as lower-limb ischemia is uncommon, with only a limited number of such cases reported in the literature. This report aims to describe an atypical clinical presentation of aortic dissection characterized by pain in a lower limb.

## Introduction

Type A aortic dissection is a surgical emergency that requires immediate management [1]. While it usually presents with chest pain radiating to the interscapular region, it can also manifest with atypical symptoms [2]. Among the complications resulting from aortic dissection, malperfusion is particularly severe. Malperfusion syndrome is characterized by ischemia of an organ or limb caused by compression of the true lumen due to the dissection. Acute type A aortic dissection-related lower-limb malperfusion is usually reversed after timely proximal aortic repair. This report describes a clinical case of type A aortic dissection with an atypical presentation.

## Case presentation

A 65-year-old man presented to the emergency department of Charleroi University Hospital on his own due to sudden pain that appeared in the morning in his left lower limb. His medical history included hypertension, dyslipidemia, and a left middle cerebral artery (MCA) stroke in 2010. On physical examination, his vital signs were within normal limits - heart rate: 81 beats/minute, respiratory rate: 21 breaths/minute, oxygen saturation on room air: 97%, temperature: 36.4 °C, blood pressure: 143/65 mmHg in the left upper limb and 151/67 mmHg in the right upper limb.

On clinical examination, the patient was agitated, confused, and uncomfortable. Neurologically, there was left lower facial paralysis, left-sided hemiplegia, and hypoesthesia of the left lower limb. Vascular examination revealed that the left lower limb was warm and pale, with a capillary refill time greater than three seconds. No pulse was detected in the left femoral, popliteal, or dorsalis pedis arteries, while upper extremity pulses were intact. The initial blood test results showed no abnormalities and were within normal limits (Table 1).

**Table 1:** Laboratory results at admission eGFR: estimated glomerular filtration rate; MDRD: Modification of Diet in Renal Disease; AST: aspartate aminotransferase; GOT: glutamic oxaloacetic transaminase; ALT: alanine aminotransferase; GPT: glutamic-pyruvic transaminase

Test	Result	Reference range
Hemoglobin	15.4 g/dL	13.0–17.0
White blood cells	7090 /μL	4 000–10 000
Neutrophils	3400 /μL	2 000–7 000
Lymphocytes	2930 /μL	1 000–3 000
Monocytes	580 /μL	200–1 000
Platelets	152 000 /μL	150 000–420 000
C-reactive protein (CRP)	<1 mg/L	<5
Prothrombin time (PT)	100%	70–120
International normalized ratio (INR)	1	0.8–1.2
Activated partial thromboplastin time (APTT)	22 seconds	28–40
Fibrinogen	2.3 g/L	2.0–4.5
Sodium	142 mmol/L	136–145
Potassium	3.5 mmol/L	3.5–5.1
Chloride	108 mmol/L	98–107
Bicarbonate	18 mmol/L	23–31
Urea	30 mg/dL	18–55
Creatinine	1.13 mg/dL	0.72–1.25
eGFR (MDRD)	65 mL/min	>60
Total bilirubin	0.93 mg/dL	0.3–1.1
Alkaline phosphatase	53 U/L	40–150
Gamma-glutamyl transferase (GGT)	77 U/L	12–64
AST (GOT)	26 U/L	5–34
ALT (GPT)	30 U/L	<55
Lipase	46 U/L	8–78
Troponin	0.006 ng/mL	<0.030

**Figure 1:**
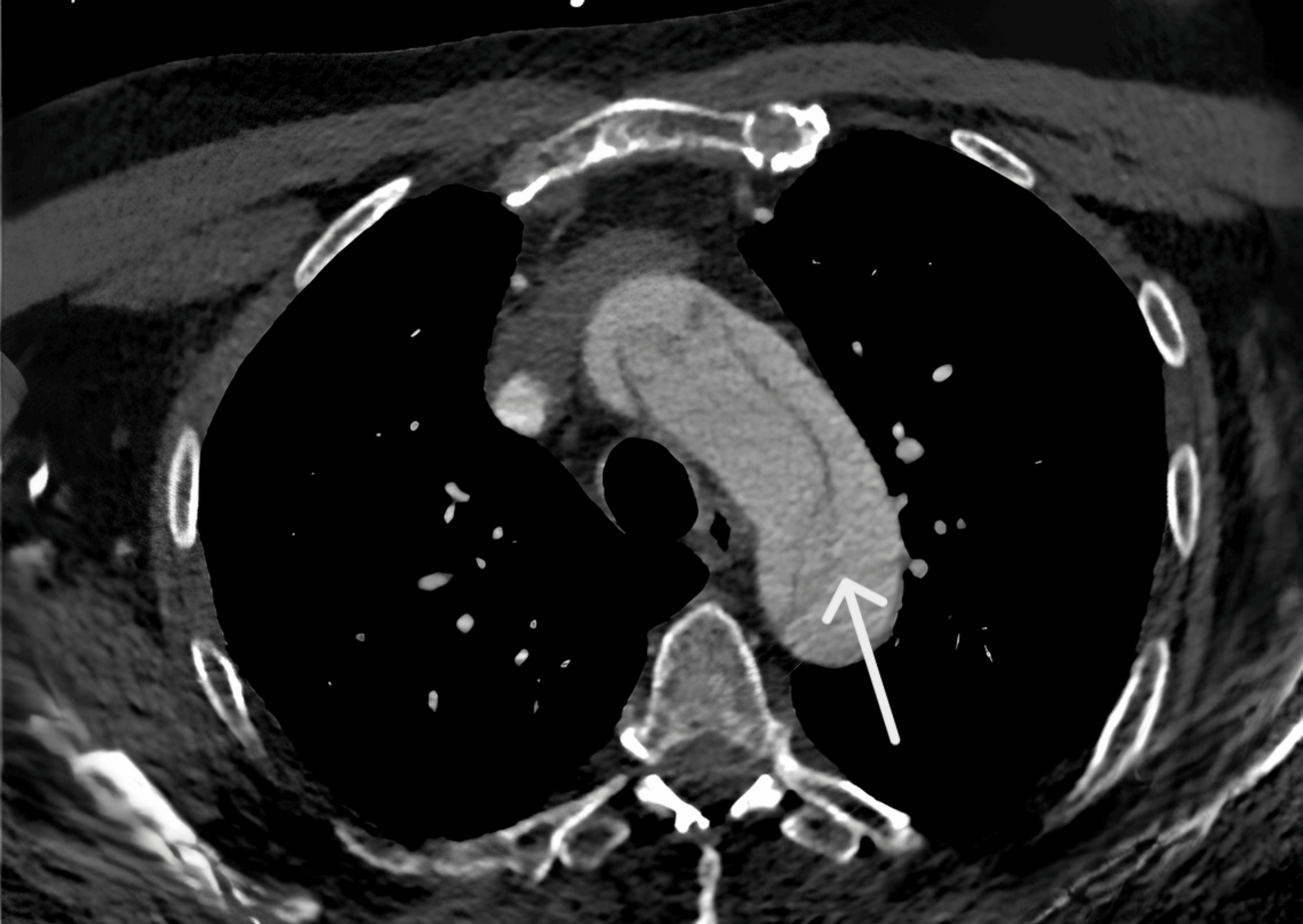
Axial CT image showing Stanford type A aortic dissection CT: computed tomography

CT angiography of the aorta showed a Stanford type A aortic dissection starting from the base of the ascending aorta at the valvular plane (Figure 1) [3]. In the abdominal region, the false lumen caused hypoperfusion of the posterior third of the left kidney, while the true lumen supplied the right iliac bifurcation. The patient underwent surgery consisting of resection of the ascending aorta and aortic arch with interposition of a Dacron graft under selective extracorporeal circulation. Immediately post-intervention, examination revealed that pulses in the left lower limb were restored, its color had normalized, and capillary refill time returned to less than two seconds.

The postoperative course included a seven-day stay in the ICU. Postoperatively, persistent bleeding occurred at the wound suture sites, leading to hemorrhagic shock and requiring surgical re-intervention. The patient also developed cellulitis on the inner aspect of the left thigh, which was treated with piperacillin-tazobactam antibiotic therapy. After 14 days of hospitalization, the patient was discharged home under close follow-up, without any sequelae.

## Discussion

Aortic dissection is defined as a longitudinal cleavage of the aortic wall at the level of the media. The underlying pathophysiological process involves an intimal tear and the formation of two channels, known as the true and false lumens, caused by blood flow entering the aortic wall [3]. There are several classifications of aortic dissection, including the Stanford classification, the DeBakey classification, and the European Society of Cardiology classification. The Stanford classification is a therapeutic system based entirely on surgical indications and remains the most widely used in clinical practice.

Non-modifiable risk factors for aortic dissection include advanced age, male sex, the presence of an aortic aneurysm, Marfan syndrome, and inflammatory diseases such as Takayasu arteritis. Modifiable risk factors include hypertension (present in 65-75% of cases), smoking, intravenous drug use, and the consumption of cocaine or fluoroquinolones [3-4]. The most consistent symptom, present in about 90% of patients, is an intense, tearing chest pain. The location of the pain depends on the type of dissection. In rare cases, aortic dissection may extend to the femoral and iliac arteries, in which case patients typically experience pain in the lower limbs.

On clinical examination, findings may include a decrescendo diastolic murmur of aortic regurgitation, blood pressure asymmetry greater than 20 mmHg, absence of peripheral pulses, or neurological deficits. A hypertensive peak is more frequently observed in type B dissections (70%) and in 25-35% of type A cases [5]. Complications of aortic dissection result from the anatomical progression of the false lumen. They may manifest as limb ischemia, myocardial ischemia (20%), neurological deficits such as stroke (10-40% of type A dissections), or shock with vascular collapse due to aortic rupture or cardiac tamponade. CT angiography of the thorax is considered the gold standard for diagnosing aortic dissection, especially in emergencies where MRI or transthoracic echocardiography is not feasible or available [6].

Therapeutic management depends on the Stanford type of dissection. The main goal of medical treatment is to reduce shear stress on the aortic wall. Target values include a systolic blood pressure between 100 and 120 mmHg and a heart rate around 60 bpm, typically achieved with beta-blockers [7]. The principle of surgical treatment is the replacement of the dissected ascending aorta, with correction of any associated aortic regurgitation when necessary [8].

General review of the literature on aortic dissection presenting with limb ischemia

Malperfusion syndrome is characterized by ischemia of an organ or limb resulting from compression of the true lumen caused by the dissection [9]. The differential diagnosis of acute lower limb pain is broad and includes electrolyte imbalances, compartment syndrome, radiculopathies, cellulitis, embolism, thrombosis, and other vascular disorders.

Overall, cases of lower limb ischemia caused by aortic dissection are rare. A review of the literature involving more than 1,700 cases of aortic dissection showed that 10% of patients presented with symptoms affecting the lower limbs. Among these, only 10 cases involved isolated lower limb involvement as the initial complaint. Consequently, cases of aortic dissection may easily go unnoticed in patients who do not present with the typical associated symptoms, such as chest pain [7,10].

## Conclusions

Type A aortic dissection associated with malperfusion syndrome carries a higher mortality rate than isolated type A aortic dissection. Despite advances in medical imaging and surgical techniques, postoperative outcomes in patients with aortic dissection complicated by malperfusion syndrome remain poor. Therefore, when evaluating a patient with acute, non-traumatic lower-limb ischemia, aortic dissection should be considered in the differential diagnosis, even when classic symptoms such as chest or back pain are absent.
